# Basal body structure and cell cycle-dependent biogenesis in *Trypanosoma brucei*

**DOI:** 10.1186/s13630-016-0023-7

**Published:** 2016-02-08

**Authors:** Sue Vaughan, Keith Gull

**Affiliations:** 1 Department of Biological and Medical Sciences, Faculty of Health and Life Science, Oxford Brookes University, Oxford, OX3 0BP UK; 2 Sir William Dunn School of Pathology, University of Oxford, South Parks Road, Oxford, OX1 3RE UK

**Keywords:** Basal body, Centriole, Flagellum, Cilium, Flagellar pocket, Microtubules, Axoneme, *Trypanosoma brucei*

## Abstract

Basal bodies are microtubule-based organelles that assemble cilia and flagella, which are critical for motility and sensory functions in all major eukaryotic lineages. The core structure of the basal body is highly conserved, but there is variability in biogenesis and additional functions that are organism and cell type specific. Work carried out in the protozoan parasite *Trypanosoma brucei* has arguably produced one of the most detailed dissections of basal body structure and biogenesis within the context of the flagellar pocket and associated organelles. In this review, we provide a detailed overview of the basic basal body structure in *T. brucei* along with the accessory structures and show how basal body movements during the basal body duplication cycle orchestrate cell and organelle morphogenesis. With this in-depth three-dimensional knowledge, identification of many basal body genes coupled with excellent genetic tools makes it an attractive model organism to study basal body biogenesis and maintenance.

## The organism

The trypanosomes are a group of protozoa characterised by their possession of a single flagellum and a mass of mitochondrial DNA organised into a kinetoplast, which is connected to the proximal end of the basal bodies [1, 2]. They are members of the Excavata; Phylum Euglenozoa; Class Kinetoplastida; Order Trypansomatida; Family Trypanosomatidae; Genus *Trypanosoma*. The *T. brucei* group includes three subspecies: the zoonotic/human parasites *T. b. rhodesiense* and *T. b. gambiense* and the animal parasite *T. b. brucei*. The African trypanosomes are closely related to the South American *Trypanosoma cruzi* and the global *Leishmania* parasites [3]. However, *Leishmania* and *T. cruzi* produce an amastigote form with a shortened flagellum that appears to have a mainly sensory role. Moreover, this amastigote flagellum bears the same collapsed radial symmetry of the microtubule axoneme (9 + 0, 9v) as the primary cilium of mammalian cells [4, 5].

## Basic basal body structure

**Fig. 1:**
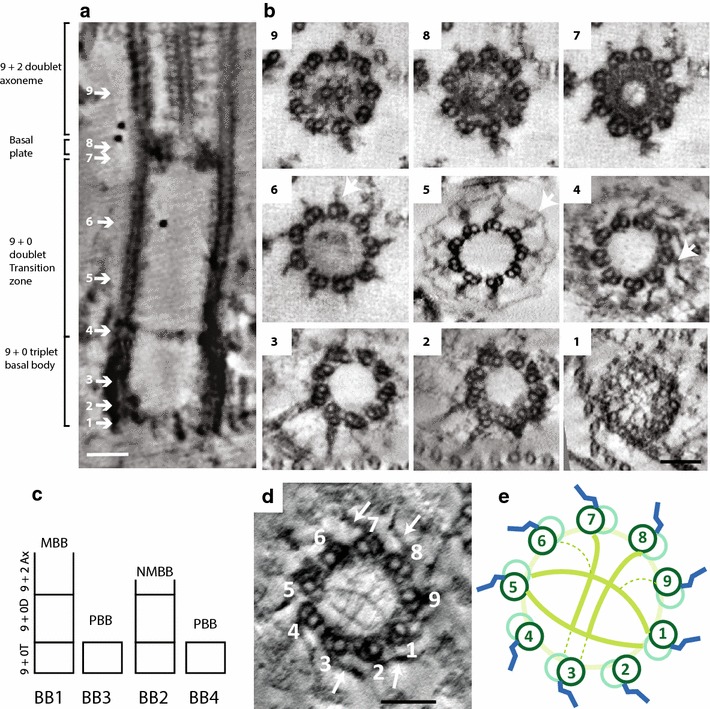
Ultrastructure of the *T. brucei* basal body. **a** Longitudinal TEM section through the entire basal body; b1–9 TEM cross sections through the mature basal body from the proximal end (**b**1) to the distal end (**b**9). See text for explanation. **c** Basal body nomenclature during basal body duplication. Mature basal body (MBB) called BB1, Newly mature basal body (NMBB) called BB2 and was formerly the pro-basal body, new pro-basal body (PBB) BB3 assembles close to BB1, new pro-basal body (PBB) BB4 assembles close to BB2; **d** transverse cross section from a tomogram illustrating the acorn structure in the lumen of the transition zone. Transitional fibres marked with *arrows* and microtubule doublets are numbered; **e** cartoon representation of a cross section through the transition zone showing the luminal acorn structure. Images were produced from work in the labs of SV and KG. The cells were detergent-extracted cytoskeletons, prepared as per method in [41]. *Scale bars* 100 nm

The *Trypanosoma brucei* exhibits a single flagellum that is attached along the length of the cell and exits the cell body via a flagellar pocket, which is located towards the posterior end of the cell [1, 6, 7]. The flagellum remains assembled throughout the cell division cycle and a new flagellum assembles alongside the old. At the start of the cell cycle, a basal body pair, mature basal body BB1 and pro-basal body BB2 (Fig. 1c) is positioned at the base of the flagellar pocket and only the mature basal body has an assembled flagellum. The mature basal body is composed of two sections; a 9 + 0 triplet arrangement of microtubules (A-, B- and C-tubules) at the proximal end of the basal body (Figs. 1b1, 2) and a transition zone, composed of a 9 + 0 arrangement of doublet microtubules. The distal end of the mature basal body is capped by a basal plate and is followed by a classical 9 + 2 microtubule axoneme (Fig. 1a) [8, 9]. The pro-basal body is composed only of 9 + 0 triplet microtubules and does not acquire a transition zone or axoneme until the cell cycle after it was formed [10]. A portion of the 9 + 0 triplet microtubule section of both the mature basal body and pro-basal body contains 5–6 cartwheel stacks at the very proximal end. These stacks are present throughout the cell cycle (Fig. 1b1) (SV/KG; unpublished).

**Fig. 2:**
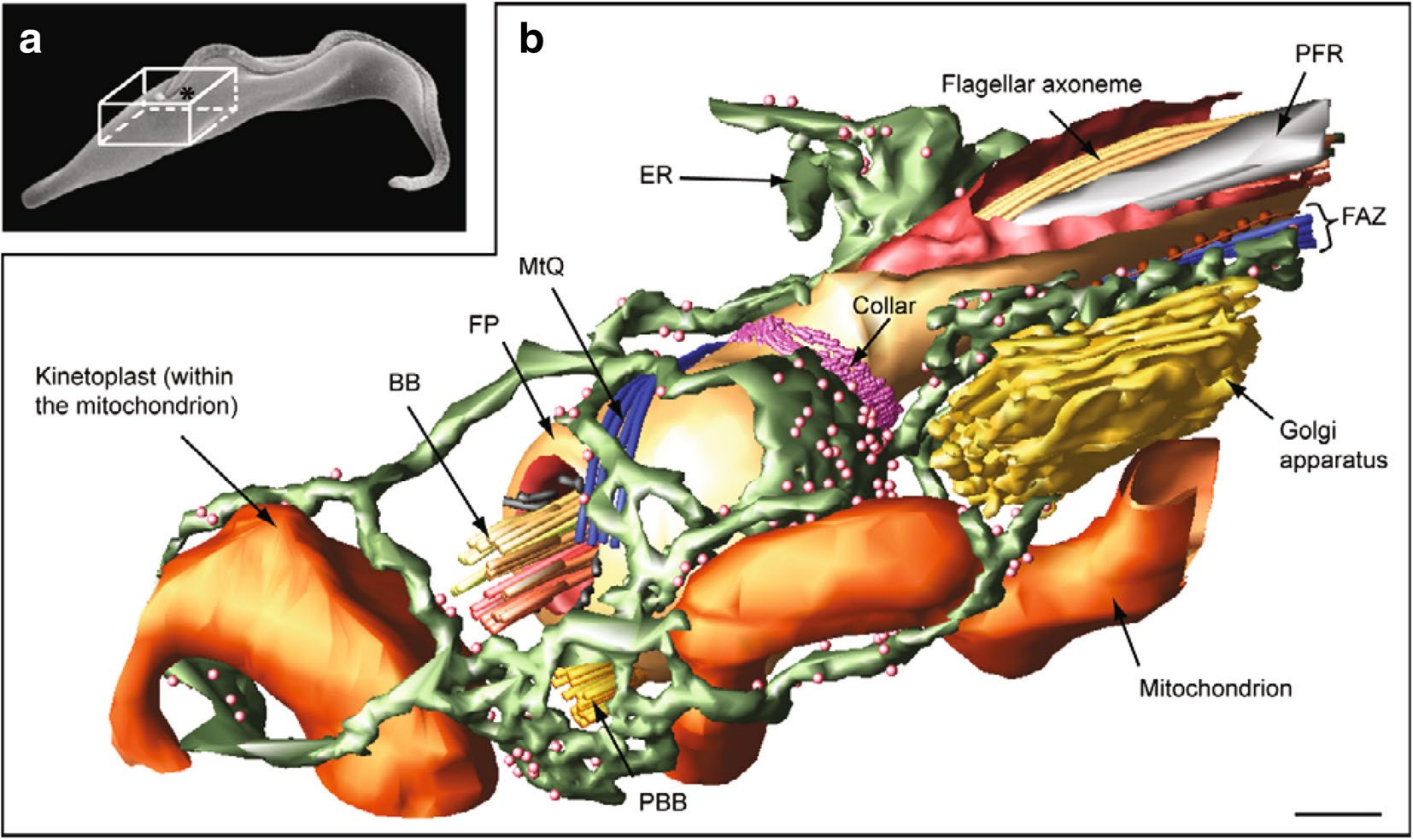
Flagellar pocket architecture of *T. brucei*.** a** Scanning electron micrograph illustrates the position of the flagellar pocket region; **b** 3D segmentation from a serial tomogram illustrates the relationship of the cytoskeletal and membrane structures associated with the pocket. *BB* basal body, *PBB* pro-basal body, *FP* flagellar pocket, *PFR* paraflagellar rod, *MtQ* microtubule quartet, *FAZ* flagellum attachment zone, *ER* endoplasmic reticulum. Scale bars 200 nm. Reprinted from [7]

The transition zone is ~400 nm long and is composed of a doublet arrangement of 9 + 0 microtubules. At the boundary between the triplet 9 + 0 microtubule portion of the basal body and the transition zone, there are a set of transitional fibres, which assemble between the A- and B-tubules of each of the nine doublet microtubules (Fig. 1b4, c; arrow) and these connect with the base of the flagellar pocket membrane [7]. In the lumen of this boundary region, where the transitional fibres are located, there is an acorn structure, which was first described in *C. reinhardtii* and other flagellated organisms and is a centrin-containing structure [11, 12] (Fig. 1d, e). The rest of the rather long transition zone lumen appears devoid of other organised filamentous structures until the basal plate is reached. Y-linkers project from between the A- and B-tubules of each of the nine doublets throughout the transition zone (Fig. 1b5, 6; arrow) except where the transitional fibres are attached at the very proximal end of the transition zone (Fig. 1b4). Detergent-extracted cytoskeletons allow the visualisation of an additional structure encircling the proximal portion of the transition zone which connects to the Y-linkers (Fig. 1b5; arrow). This cytoskeletal structure is most likely the collarette which is external to the flagellar membrane surrounding the proximal portion of the transition zone [7]. The distal end of the mature basal body is capped by a ~30 nm basal plate; a stack of two rings which contain an outer electron dense ring and an inner electron-lucent lumen. The minus ends of the central pair microtubules are embedded in the distal ring [13] (Figs. 1b7, 8). The A- and B-tubules of the transition zone extend further and the central pair microtubules extend from the basal plate together forming the 9 + 2 microtubule axoneme (Fig. 1b9). Correlations of protein components of the *T. brucei* basal body and structures described here are dealt with below. However, it is useful to note that trypanosomes have a full complement of the alpha, beta, gamma and delta tubulin gene family. The trypanosome was also one of the first organisms shown to contain epsilon tubulin and was where the canonical zeta tubulin was identified [14].

## Additional and accessory basal body structures

The trypanosome basal bodies form a master organiser for the surrounding cytoskeleton, membranous structures and organelles (Fig. 2). Hence they have a variety of appendages and accessory structures performing roles in the orchestration of cell architecture in addition to those defining basal body/pro-basal body connections for biogenesis and inheritance. Cellular electron tomography studies have highlighted the three-dimensional organisation of accessory basal body structures located on the 9 + 0 triplet microtubule portion in *T. brucei* [7]. These consist of a set of striated fibres assembled onto the mature basal body with three striated fibres (SF1, 2, 3) associated with the C-tubule of triplet five and two striated fibres associated with the C-tubule of triplet 6 (SF4, 5) [7]. A subset of these striated fibres connects to four rootlet microtubules (the microtubule quartet (MtQ)) [15], which originate between the mature basal body and pro-basal body and wrap around the flagellar pocket before inserting into the subpellicular microtubule cytoskeleton (Fig. 2). Electron tomography of cytoskeletal preparations has revealed that there are fine filament connections between the MtQ and the pro-basal body (SV/KG; unpublished).

In trypanosomes, the basal bodies serve another function in ensuring inheritance of the mitochondrial genome. The kinetoplast is physically connected to the proximal end of both the mature basal body and pro-basal body [2] (Fig. 2) by the tripartite attachment complex (TAC). The TAC represents a set of filaments connecting from the proximal end of each basal body to a specialised area of the outer mitochondrial membrane with a further set of unilateral filaments connecting between the inner mitochondrial membrane and the mitochondrial DNA [16]. This physical connection remains intact throughout cell division and the kinetoplast DNA is replicated in a periodic S phase coincident with basal body duplication [17, 18]. Basal body segregation ensures inheritance of the duplicated mitochondrial DNA to the two daughter cells [2]. There must be extensive re-organisation of the tripartite attachment complex during basal body biogenesis, but how this orchestrated and regulated is not yet clear.

## Basal body origins

Trypanosome basal bodies are always associated with a flagellum and their biogenesis, maturation and segregation are linked into the flagellum cycle. There is no encysted or other stage in the life cycle where the basal bodies are reduced to the status of centrioles nor one where the structures are removed entirely as in some amoeba-flagellate organisms such as *Physarum* or *Naegleria* [19, 20]. Importantly, one should also note that the basal bodies are never associated with the poles of the spindle or the nuclear envelope. The basal bodies act as master organisers of the cytoplasm, organelle position and cell shape without direct involvement with the nucleus and spindle poles. An intranuclear, fibrous, non-membrane associated, spindle organising centre was identified in a TEM study of mitosis [21]. However, no further ultrastructural or biochemical characterisation has been carried out and it is noteworthy to add that no basal body proteins have been reported to localise to the poles of the mitotic spindle (SV/KG; personal communication).

## Basal body life cycle and other functions

**Fig. 3:**
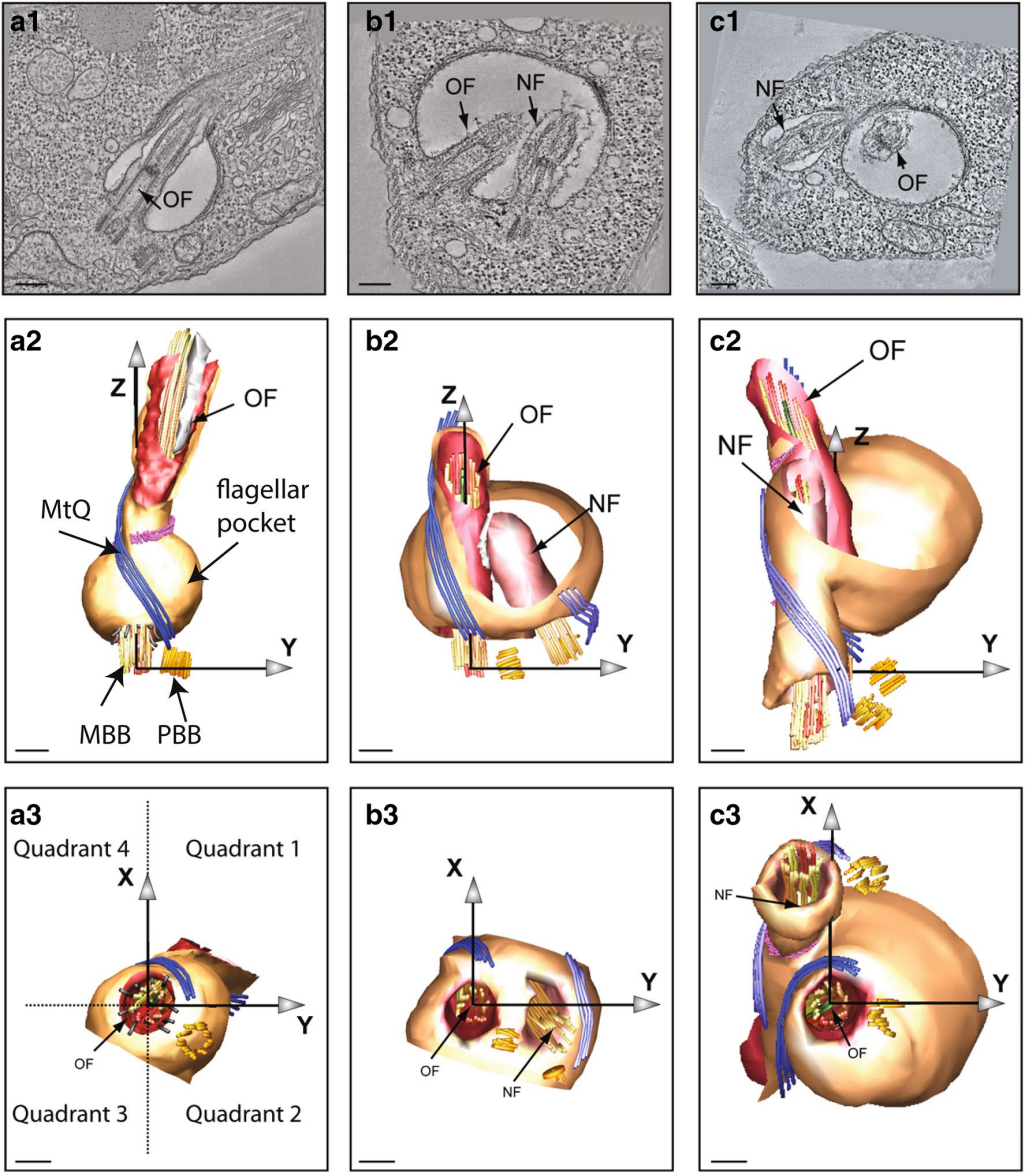
Rotation of the newly mature basal body around the old flagellum in *T. brucei.* a–c Representative tomograms showing different stages of basal body and flagellar pocket morphogenesis; a1,b1,c1 slices through the flagellar pockets from the original tomograms; Tomogram models (a2–c3) contain Cartesian axes previously described that are used to map and describe the rotation [7]; a2,a3 Two views of the model of a tomogram illustrating a cell in which the pro-basal body (PBB; BB2) is located on the bulge side of the flagellar pocket in quadrant 2 prior to the start of the cell cycle; b2–b3: In this cell, the pro-basal body (BB2) has matured and has subtended a new flagellum (NF) that has invaded the existing flagellar pocket and connected to the old flagellum (OF). The new flagellum is still positioned essentially as in a2–a3: quadrant 2; c2,c3 A later stage in the cell cycle just before flagellar pocket division. The new flagellum (NF) is now in a more posterior location and lies in quadrant 4. Thus, a rotation of the newly mature basal body (BB2) has occurred around the old flagellum within the confines of the existing flagellar pocket. Please see text for explanation of the rotation. Mature basal body (MBB); pro-basal body (PBB); Two new pro-basal bodies (BB3 and BB4) in yellow. Microtubule Quartet (MtQ). Scale bars: 200 nm. Reprinted from [25]

Basal body biogenesis in *T. brucei* exhibits many of the features of mammalian centrioles with maturation, duplication and segregation phases. During the cell division cycle, the microtubule subpellicular cytoskeleton of the cell remains intact but new microtubules are added such that two daughters are produced [22]. The trypanosome basal body pair is constantly associated with a flagellar pocket [6]. This flagellar pocket represents, perhaps, the most iconic of privileged membrane transport sites conjectured to be associated with most cilia or flagella [23]. In trypanosomes, this example is highly obvious since, given the subpellicular microtubule corset, it represents the sole site for endocytosis and exocytosis in the cell [24]. Electron tomography of the flagellar pocket area has highlighted its complexity and demonstrated that a new flagellar pocket forms from the existing flagellar pocket during the cell cycle [7, 25]. Basal body duplication and segregation are, therefore, highly coordinated with both flagellar pocket biogenesis and mitochondrial DNA replication. At the start of the cell cycle, the mature basal body (BB1) (Fig. 1c) and pro-basal body (BB2) are positioned parallel to one another and the mature basal body subtends a single attached flagellum, which remains assembled throughout the cell cycle. The first morphological indicators of cell cycle initiation at G1/S transition are extension of a transition zone on the pro-basal body (BB2) and growth of a new MtQ [10, 25] (Fig. 3a1–3). The newly maturing basal body (BB2) invades a specific portion of the existing flagellar pocket and two new pro-basal bodies (BB3 and BB4) form orthogonal to each now mature basal body (BB1 and BB2) (Fig. 3b1–3). A nascent new flagellar pocket forms within the confines of the old flagellar pocket as the new flagellum elongates. There is an anti-clockwise rotation of the new mature basal body (BB2) around the old flagellum (Fig. 3c1–3) and this movement is very likely to be critical for flagellar pocket morphogenesis (Fig. 3c1–3). The two new pro-basal bodies (BB3 and BB4) must also re-orientate to a parallel position relative to each mature basal body and unpublished work in our labs shows that this occurs prior to mitosis (SV/KG; unpublished).

## Identification of basal body components

Centrioles and basal bodies are structurally highly conserved in the core ninefold symmetrical microtubule and cartwheel structure. However, many differences remain in accessory structures and additional specialised functions in diverse cell types. Bioinformatics analysis of 45 eukaryotic organisms revealed 14 core ancestral centriole proteins and 12 of these (δ−tubulin, centrin 2, WDR16, SAS-4, SAS-6, POC1, CEP164, DIP13, VFL1, CEP76, POC5) are conserved in *T. brucei,* but no orthologues were identified for CEP135 or Centriolin [26]. CEP135/Bld10 localises to a portion of the cartwheel spokes close to the triplet microtubules in *C. reinhardtii* and knockdown leads to shortened cartwheel spokes [27], but it seems unlikely to be part of the spokes themselves, due to the lack of conservation. Both the structure of the cartwheel and function of SAS-6 are conserved in *T. brucei* and *L. major* [28, 29]. Proteomic analysis of detergent and salt-extracted flagella preparations of *T. brucei,* which includes the basal body and pro-basal body, identified 331 proteins of which 208 are trypanosomatid-specific. This proteome identified many putative and confirmed ciliopathy genes in the proteome including 34 genes mapping to 25 loci where ciliary dysfunction has been recorded [30]. Other proteomic analysis of *T. brucei* flagella has extended this list and validated many of the proteome constituents, including intact isolated flagella with membranes [31] and enriched flagella membrane fractions, which both contain a small number of basal body proteins [32].

## Notable basal body findings

The trypanosome flagella have proven extraordinarily useful for studies on basal body functions as well as flagella-associated functions. Specific basal body insights have come in the involvement of γ-tubulin in basal body nucleation of the central pair [9]; the discovery of ζ-tubulin [14] whose importance is now revealed [33]; the use of proximal end functions in organelle segregation (mitochondrion/kinetoplast) [2]; positional information in cell form acquisition, specific inheritance patterns of basal bodies [25] and the flagellar pocket cytoarchitecture [7, 34, 35].

## Strengths and future of basal body research in *T. brucei*

One of the distinct advantages of working with *T. brucei* in basal body research is that each basal body and pro-basal body can be unequivocally identified within a cell during cell division and differentiation to distinct cell types. The maintenance of an old flagellum and the formation of a new flagellum alongside in the same cell offer unique opportunities to study assembly and turnover of flagellar components. Cultured *T. brucei* cells are very easy to grow and quick to generate mutant cell lines. There are very well-developed tools to allow the molecular dissection of basal body functions in *T. brucei*. These include inducible RNAi, allowing stable cell lines to be generated for lethal genes [36–38] and a genome-wide RNAi study identified lethal and non-lethal genes for the two main life cycle forms and during differentiation [39]. There are also excellent tools for PCR tagging at the endogenous locus by homologous recombination, allowing one to go from PCR amplification to transfection into cells within a day [40]. Indeed, there is a whole-genome tagging project underway, which will undoubtedly identify numerous basal body genes. These unique features, phylogenetic positioning and tools make it an attractive model to study basal body biogenesis.

## References

[1] Vickerman K (1969). On the surface coat and flagellar adhesion in trypanosomes. J Cell Sci.

[2] Robinson DR, Gull K (1991). Basal body movements as a mechanism for mitochondrial genome segregation in the trypanosome cell cycle. Nature.

[3] Vickerman K (1994). The evolutionary expansion of the trypanosomatid flagellates. Int J Parasitol.

[4] Gluenz E, Höög JL, Smith AE, Dawe HR, Shaw MK, Gull K (2010). Beyond 9+ 0: noncanonical axoneme structures characterize sensory cilia from protists to humans. FASEB J..

[5] Wheeler RJ, Gluenz E, Gull K (2013). The limits on trypanosomatid morphological diversity. PLoS ONE.

[6] Vickerman K (1969). The fine structure of Trypanosoma congolense in its bloodstream phase. J protozoology.

[7] Lacomble S, Vaughan S, Gadelha C, Morphew MK, Shaw MK, McIntosh JR (2009). Three-dimensional cellular architecture of the flagellar pocket and associated cytoskeleton in trypanosomes revealed by electron microscope tomography. J Cell Sci.

[8] Vaughan S, Shaw M, Gull K (2006). A post-assembly structural modification to the lumen of flagellar microtubule doublets. Curr biol.

[9] McKean PG, Baines A, Vaughan S, Gull K (2003). Gamma-tubulin functions in the nucleation of a discrete subset of microtubules in the eukaryotic flagellum. Curr biol.

[10] Sherwin T, Gull K (1989). The cell division cycle of Trypanosoma brucei brucei: timing of event markers and cytoskeletal modulations. Philos Trans R Soc Lond B Biol Sci.

[11] Geimer S, Melkonian M (2004). The ultrastructure of the *Chlamydomonas reinhardtii* basal apparatus: identification of an early marker of radial asymmetry inherent in the basal body. J Cell Sci.

[12] Geimer S, Melkonian M (2005). Centrin scaffold in *Chlamydomonas reinhardtii* revealed by immunoelectron microscopy. Eukaryot Cell.

[13] Hoog JL, Lacomble S, O’Toole ET, Hoenger A, McIntosh JR, Gull K (2014). Modes of flagellar assembly in *Chlamydomonas reinhardtii* and Trypanosoma brucei. Elife..

[14] Vaughan S, Attwood T, Navarro M, Scott V, McKean P, Gull K (2000). New tubulins in protozoal parasites. Curr biol.

[15] Godfrey DG, Taylor AE, Lanham SM (1970). Studies on the biology of trypanosomes with special reference to their surface properties. Trans R Soc Trop Med Hyg.

[16] Ogbadoyi EO, Robinson DR, Gull K (2003). A high-order trans-membrane structural linkage is responsible for mitochondrial genome positioning and segregation by flagellar basal bodies in trypanosomes. Mol Biol Cell.

[17] Cosgrove WB, Skeen MJ (1970). The cell cycle in *Crithidia fasciculata*. Temporal relationships between synthesis of deoxyribonucleic acid in the nucleus and in the kinetoplast. The. J Protozool.

[18] Woodward R, Gull K (1990). Timing of nuclear and kinetoplast DNA replication and early morphological events in the cell cycle of *Trypanosoma brucei*. J Cell Sci.

[19] Fulton C, Dingle AD (1971). Basal bodies, but not centrioles. Naegleria J Cell Biol.

[20] Havercroft JC, Gull K (1983). Demonstration of different patterns of microtubule organization in Physarum polycephalum myxamoebae and plasmodia using immunofluorescence microscopy. Eur J Cell Biol.

[21] Ogbadoyi E, Ersfeld K, Robinson D, Sherwin T, Gull K (2000). Architecture of the Trypanosoma brucei nucleus during interphase and mitosis. Chromosoma.

[22] Sherwin T, Gull K (1989). Visualization of detyrosination along single microtubules reveals novel mechanisms of assembly during cytoskeletal duplication in trypanosomes. Cell.

[23] Benmerah A (2013). The ciliary pocket. Curr Opin Cell Biol.

[24] Field MC, Adung’a V, Obado S, Chait BT, Rout MP (2012). Proteomics on the rims: insights into the biology of the nuclear envelope and flagellar pocket of trypanosomes. Parasitology.

[25] Lacomble S, Vaughan S, Gadelha C, Morphew MK, Shaw MK, McIntosh JR (2010). Basal body movements orchestrate membrane organelle division and cell morphogenesis in *Trypanosoma brucei*. J Cell Sci.

[26] Hodges ME, Scheumann N, Wickstead B, Langdale JA, Gull K (2010). Reconstructing the evolutionary history of the centriole from protein components. J Cell Sci.

[27] Ohta T, Essner R, Ryu JH, Palazzo RE, Uetake Y, Kuriyama R (2002). Characterization of Cep135, a novel coiled-coil centrosomal protein involved in microtubule organization in mammalian cells. J Cell Biol.

[28] van Breugel M, Wilcken R, McLaughlin SH, Rutherford TJ, Johnson CM (2014). Structure of the SAS-6 cartwheel hub from Leishmania major. Elife..

[29] Hu H, Liu Y, Zhou Q, Siegel S, Li Z (2015). The centriole cartwheel protein SAS-6 in trypanosoma brucei is required for probasal body biogenesis and flagellum assembly. Eukaryot Cell.

[30] Broadhead R, Dawe HR, Farr H, Griffiths S, Hart SR, Portman N (2006). Flagellar motility is required for the viability of the bloodstream trypanosome. Nature.

[31] Subota I, Julkowska D, Vincensini L, Reeg N, Buisson J, Blisnick T (2014). Proteomic analysis of intact flagella of procyclic Trypanosoma brucei cells identifies novel flagellar proteins with unique sub-localization and dynamics. Mol Cell Proteom.

[32] Oberholzer M, Langousis G, Nguyen HT, Saada EA, Shimogawa MM, Jonsson ZO, Nguyen SM, Wohlschlegel JA, Hill KL (2011). Independent analysis of the flagellum surface and matrix proteomes provides insight into flagellum signaling in mammalian-infectious Trypanosoma brucei. Mol Cell Proteomics.

[33] Turk E, Wills AA, Kwon T, Sedzinski J, Wallingford JB, Stearns T (2015). Zeta-Tubulin is a member of a conserved tubulin module and is a component of the centriolar basal foot in multiciliated cells. Curr Biol.

[34] Absalon S, Blisnick T, Bonhivers M, Kohl L, Cayet N, Toutirais G (2008). Flagellum elongation is required for correct structure, orientation and function of the flagellar pocket in *Trypanosoma brucei*. J Cell Sci.

[35] Absalon S, Kohl L, Branche C, Blisnick T, Toutirais G, Rusconi F (2007). Basal body positioning is controlled by flagellum formation in *Trypanosoma brucei*. PLoS ONE.

[36] LaCount DJ, Bruse S, Hill KL, Donelson JE (2000). Double-stranded RNA interference in *Trypanosoma brucei* using head-to-head promoters. Mol Biochem Parasitol.

[37] Bastin P, Ellis K, Kohl L, Gull K (2000). Flagellum ontogeny in trypanosomes studied via an inherited and regulated RNA interference system. J Cell Sci.

[38] Wang Z, Morris JC, Drew ME, Englund PT (2000). Inhibition of *Trypanosoma brucei* gene expression by RNA interference using an integratable vector with opposing T7 promoters. J Biol Chem.

[39] Alsford S, Turner DJ, Obado SO, Sanchez-Flores A, Glover L, Berriman M (2011). High-throughput phenotyping using parallel sequencing of RNA interference targets in the African trypanosome. Genome Res.

[40] Dean S, Sunter J, Wheeler RJ, Hodkinson I, Gluenz E, Gull K (2015). A toolkit enabling efficient, scalable and reproducible gene tagging in trypanosomatids. Open Biol..

[41] Hoog JL, Gluenz E, Vaughan S, Gull K (2010). Ultrastructural investigation methods for Trypanosoma brucei. Methods Cell Biol.

